# 静脉蔗糖铁治疗复发性缺铁性贫血的疗效和安全性

**DOI:** 10.3760/cma.j.issn.0253-2727.2023.05.009

**Published:** 2023-05

**Authors:** 京倩 刘, 夏婉 杨, 旭 刘, 靖 胡, 向荣 胡, 小霞 李, 雨霏 赵, 忆萌 史, 保航 张, 文睿 杨, 广新 彭, 馨 赵, 凤奎 张

**Affiliations:** 中国医学科学院血液病医院（中国医学科学院血液学研究所），实验血液学国家重点实验室，国家血液系统疾病临床医学研究中心，细胞生态海河实验室，天津 300020 State Key Laboratory of Experimental Hematology, National Clinical Research Center for Blood Diseases, Haihe Laboratory of Cell Ecosystem, Institute of Hematology & Blood Diseases Hospital, CAMS & PUMC, Tianjin 300020, China

**Keywords:** 贫血，缺铁性, 复发, 静脉铁剂, 蔗糖铁, Anemia, iron deficiency, Recurrence, Intravenous iron supplementation, Sucrose iron

## Abstract

**目的:**

评估静脉铁剂治疗复发性缺铁性贫血（IDA）的有效性和安全性。

**方法:**

回顾性分析2012年5月至2021年12月期间90例复发性IDA患者的临床资料，比较静脉铁剂组与口服铁剂组的疗效及不良反应发生情况。

**结果:**

90例复发性IDA患者中，男20例，女70例，中位年龄40（14，85）岁。60例接受静脉铁剂治疗，30例接受口服铁剂治疗。补铁治疗后第4、8周，静脉铁剂组血液学反应率分别为80.0％（48/60）、96.7％（58/60），显著高于口服铁剂组患者的3.3％（1/30）、46.7％（14/30）（Fisher，均*P*<0.001）；HGB较基线分别中位增高38（4，66）g/L和44.5（18，80）g/L，显著高于口服铁剂组的7（1，22）g/L和19（3，53）g/L（*t*＝10.720，*P*<0.001；*t*＝7.392，*P*<0.001）；HGB恢复正常患者分别为33例（55.0％）和54例（90.0％），显著高于口服铁剂组的0例和13例（43.3％）（Fisher，均*P*<0.001）。静脉铁剂组和口服铁剂组分别有26例和7例在治疗前以及治疗后8周检测了铁代谢指标。治疗后8周静脉铁剂组血清铁蛋白（SF）中位增高值113.7（49.7，413.5）µg/L，53.8％（14/26）的患者血清SF≥100 µg/L，显著高于口服铁剂组的14.0（5.8，84.2）µg/L（*t*＝4.760，*P*<0.001），而口服铁剂组则未有SF≥100 µg/L患者（Fisher，*P*＝0.013）。静脉铁剂组不良反应率为3.3％（2/60），远低于口服铁剂组20.0％（6/30）（Fisher，*P*＝0.015）。

**结论:**

复发性IDA应用静脉补铁较口服补铁治疗血液学反应率更高，HGB上升更快，储存铁补足率更高，患者耐受良好。

## Abstract (en)

**Objective:**

To evaluate the efficacy and safety of intravenous iron supplementation in patients with recurrent iron deficiency anemia（IDA）.

**Methods:**

This retrospective analysis of 90 patients with recurrent IDA from May 2012 to December 2021 was conducted, comparing the efficacy and safety of the intravenous iron therapy group and the oral iron therapy group.

**Results:**

Among the 90 patients with recurrent IDA, 20 were males and 70 were females, with a median age of 40（range: 14–85）years. A total of 60 patients received intravenous iron supplementation and 30 received oral iron supplementation. The hematologic response rates in the intravenous iron group were significantly higher than those in the oral iron group at 4 and 8 weeks after treatment［80.0％（48/60）*vs* 3.3％（1/30）and 96.7％（58/60）*vs* 46.7％（14/30）, all *P*<0.001, respectively］. The median increase in hemoglobin levels was also significantly higher in the intravenous iron group than in the oral iron group［38（4, 66）g/L *vs* 7（1, 22）g/L at week 4 and 44.5（18, 80）g/L *vs* 19（3, 53）g/L at week 8, all *P*<0.001］. The intravenous iron group had a significantly higher proportion of patients who achieved normal hemoglobin levels than the oral iron group（55.0％ *vs* 0 and 90％ *vs* 43.3％, all P<0.001, respectively）. Iron metabolism indicators were tested before and after 8 weeks of treatment in 26 and 7 patients in the intravenous and oral iron groups, respectively. The median increase in serum ferritin（SF）levels in the intravenous iron group 8 weeks after treatment was 113.7（49.7, 413.5）µg/L, and 54％（14/26）of these patients had SF levels of ≥100 µg/L, which was significantly higher than the median increase in SF levels in the oral iron group［14.0（5.8, 84.2）µg/L, *t*＝4.760, *P*<0.001］and the proportion of patients with SF levels of ≥100 µg/L（*P*＝0.013）. The incidence of adverse reactions was 3.3％（2/60）in the intravenous iron group, which was significantly lower than that in the oral iron group［20.0％（6/30）, *P*＝0.015］.

**Conclusion:**

Intravenous iron supplementation is more effective for hematologic response, faster hemoglobin increase, and higher iron storage replenishment rates compared with oral iron supplementation in patients with recurrent IDA, and it is well tolerated by patients.

缺铁性贫血（IDA）是全球最常见的贫血类型之一，与多种疾病的临床进展和死亡率增加相关。近年来，静脉蔗糖铁治疗已广泛应用于临床实践，其可显著提高患者的铁水平并改善贫血症状[1]，尽管有许多临床试验已经评估了静脉蔗糖铁治疗的有效性和安全性[2]–[4]，但关于复发性IDA的研究却非常有限。本研究旨在评估静脉蔗糖铁在复发性IDA患者中的疗效和安全性，现报道如下。

## 病例与方法

1. 病例：本研究为回顾性研究，纳入2012年5月至2021年12月于我院贫血诊疗中心诊断为复发性IDA患者共90例，其中60例由口服铁剂转换为静脉铁剂治疗，30例患者仍以口服铁剂治疗，比较两组患者铁剂治疗的疗效及安全性。复发性IDA定义为明确诊断为IDA的患者经口服铁剂治疗后，HGB恢复正常后再次或多次出现HGB下降。

2. 补铁治疗方法：静脉补铁：蔗糖铁注射液（100 mg/5 ml）用0.9％的生理盐水稀释后静脉滴注。首次100 mg，此后每次200 mg，给药频率为每周不超过3次。补铁总量（mg）＝体重（kg）×［150（g/L，男）或140（g/L，女）−目前HGB（g/L）］× 0.24+500。口服补铁：使用患者原治疗有效的口服铁剂，按照推荐标准剂量和方法，或改用右旋糖酐铁分散片每日3次，每次50 mg，连续口服治疗。

3. 疗效标准：铁剂治疗后4周、8周进行疗效评价。HGB较基线升高≥20 g/L评定为获得血液学反应，HGB未升高或升高不足20 g/L者均评定为未获血液学反应。将HGB≥120 g/L（男）或≥110 g/L（女）定义为HGB达标，即HGB恢复正常。将血清铁蛋白（SF）≥100 µg/L定义为储存铁补足，SF未升高或未达到100 µg/L者均认为储存铁未补足。

4. 统计学处理：患者数据采用SPSS 25.0软件进行统计分析。由于患者基线资料不符合正态分布，因此使用中位数（*IQR*）进行描述性统计，两组之间使用独立样本Wilcoxon检验，分类变量差异性比较使用Fisher检验。而治疗前后的配对资料也不符合正态分布，因此采用配对样本Wilcoxon检验进行分析。两组客观指标差值符合正态分布，因此采用独立样本*t*检验进行分析。*P*<0.05认为差异具有统计学意义。

## 结果

1. 基线资料：90例患者均为明确病因并经过病因治疗的患者，其中男20例（22.2％）、女70例（77.8％），中位年龄40（14，85）岁，中位HGB 76.5（32，109）g/L，中位病史长度为5（1，39）年，中位复发次数2（1，7）次。静脉铁剂组与口服铁剂组患者的基线临床特征见表1。

**表1:** 接受静脉铁剂与继续接受口服铁剂的复发性缺铁性贫血（IDA）患者基线临床特征比较

基线临床特征	静脉铁剂组（60例）	口服铁剂组（30例）	统计量	*P*值
性别（例，男/女）	13/47	7/23	Fisher	1.000
中位年龄（岁）	40（14, 74）	40.5（16, 85）	−1.070	0.284
中位HGB（g/L）	72.5（32, 108）	83（41, 109）	−2.894	0.004
中位Hct（%）	25.5（10.3, 35.3）	29.0（17.7, 37.5）	−3.273	0.001
中位MCV（fl）	69.0（57.7, 88.7）	72.3（58.4, 84.8）	−1.147	0.141
中位MCH（pg）	19.4（14.2, 28.2）	19.9（15.5, 25.5）	−1.387	0.165
中位MCHC（g/L）	279（228, 328）	284（232, 318）	−1.196	0.232
中位SF（µg/L）	3.8（1.0, 13.3）	10.3（3.2, 15.7）	−2.445	0.014
贫血程度^a^[例（%）]			−1.108	0.268
轻度	11（18.3）	10（33.3）		
中度	37（61.7）	17（56.7）		
重度	12（20.0）	3（10.0）		
中位复发次数	2（1, 6）	3（1, 7）	−3.258	0.002
中位病史（年）	3（1, 39）	11（4, 39）	−3.660	0.001
导致IDA的原因			2.509	0.313
消化道疾病	32（53.3）	11（36.7）		
妇科疾病	18（30.0）	11（36.7）		
摄入不足	10（16.7）	8（26.6）		

**注** Hct：血细胞压积；MCV：平均红细胞体积；MCH：红细胞平均血红蛋白含量；MCHC：红细胞平均血红蛋白浓度；SF：血清铁蛋白。^a^：90 g/L<HGB<120 g/L为轻度贫血，60 g/L≤HGB≤90 g/L为中度贫血，30 g/L≤HGB<60 g/L为重度贫血

2. 血液学反应：静脉铁剂组蔗糖铁用量中位数为1 400（1 000，2 200）mg。治疗后4周，静脉铁剂组血液学反应率为80.0％（48/60），明显高于口服铁剂组的3.3％（1/30）（Fisher，*P*<0.001）。至治疗后8周，静脉铁剂组与口服铁剂血液学反应率分别为96.7％（58/60）和46.7％（14/30），静脉补铁治疗疗效显著高于口服铁剂（Fisher，*P*<0.001）。补铁治疗后4周，静脉和口服补铁患者HGB均较治疗前基线升高，静脉铁剂组的HGB中位增高38（4，66）g/L，显著高于口服铁剂组的7（1，22）g/L（*t*＝10.720，*P*<0.001）。治疗后8周，静脉铁剂组HGB较基线中位增高44.5（18，80）g/L，显著高于口服铁剂组的19（3，53）g/L（*t*＝7.392，*P*<0.001）。补铁治疗后4周、8周，静脉铁剂组分别有55.0％（33/60）和90.0％（54/60）的患者HGB可达到正常水平，显著高于口服铁剂组的0和43.3％（13/30）（Fisher，均*P*<0.001）。

3. 红细胞参数变化：治疗后8周，静脉铁剂治疗组58例患者获得血液学反应，其MCV较治疗前基线值中位增加14.8（2.3，29.6）fl，MCH中位增加6.8（1.2，18.4）pg，显著高于口服铁剂组14例获得血液学反应患者的MCV［7.7（0.3，17.0）fl］（*t*＝3.307，*P*＝0.002）和MCH［4.1（0.5，8.4）pg］（*t*＝2.646，*P*＝0.011）。

4. 铁代谢参数变化：静脉铁剂组和口服铁剂组分别有26例和7例在治疗前以及治疗后8周检测了铁代谢指标。治疗前，静脉铁剂组患者中位SF为3.8（1.0，13.3）µg/L，较口服铁剂组中位SF 10.3（3.2，15.7）µg/L储存铁更低（*W*＝−2.445，*P*＝0.014）。治疗后8周，静脉铁剂组患者血清中位SF为116.9（52.8，417.3）µg/L，较治疗前明显升高（*W*＝−4.457，*P*<0.001），而口服铁剂组患者血清中位SF为19.9（17.1，88.1）µg/L，较治疗前明显升高（*W*＝−2.366，*P*＝0.018）。治疗后8周，静脉铁剂组中位血清SF较基线增加值为113.7（49.7，413.5）µg/L，显著高于口服铁剂组14.0（5.8，84.2）µg/L的中位血清SF增加值（*t*＝4.760，*P*<0.001）。治疗后8周，静脉铁剂组53.8％（14/26）患者的SF≥100 µg/L，口服铁剂组未有患者治疗后SF≥100 µg/L（Fisher，*P*＝0.013）。

5. 不良反应：静脉铁剂治疗组患者除静脉注射局部轻微疼痛外，60例患者中有2例（3.3％）发生皮肤瘙痒、皮疹，经对症处理后缓解，无胸闷、呼吸困难、低血压等严重不良反应发生。口服铁剂组30例患者中有6例（20.0％）发生消化道不良反应，包括3例便秘，2例上腹部不适，1例恶心，对症处理好转，无患者因不良反应停用口服铁剂治疗。静脉铁剂治疗组不良反应发生率显著低于口服铁剂治疗组（Fisher，*P*＝0.015）。

## 讨论

铁代谢与IDA的发病机制密切相关。对于复发性IDA患者，其肝脾骨髓内铁蛋白已被耗尽，导致红细胞所需的铁不足，进而引起血红蛋白合成异常，表现为小细胞低色素性贫血。在口服铁剂和静脉铁剂可用于IDA治疗的情况下，由于口服铁剂的安全性更高且不良反应较少，大多数情况下都是首选的治疗方法[2],[5]。口服铁治疗IDA时吸收率较低，吸收的铁首先用于合成血红蛋白，成熟红细胞逐渐从小细胞低色素状态恢复至正常，铁粒幼红细胞仍未完全恢复到正常水平，患者从IDA阶段进入缺铁性红细胞生成阶段（IDE）。只有持续补铁，铁粒幼红细胞数量才会逐渐恢复正常，但组织内的储存铁仍低于正常水平，此时患者进入缺铁（ID）阶段，仍出现其他系统缺铁的症状。为了彻底恢复组织内的铁储存，需继续口服铁剂治疗以避免再次进入IDA阶段，此时也应预防其他导致铁丢失的情况如出血、铁摄入不足等情况。口服铁剂可以通过剂量依赖方式增加血清中铁调素（Hepcidin）水平，从而降低肠道中铁的吸收[6]。而口服铁治疗需要患者血红蛋白恢复正常后继续口服铁剂4～6个月以补足体内铁储备，当IDA患者的贫血症状改善时，血清Hepcidin水平会升高，进一步减缓胃肠道的铁吸收，使得IDA患者的口服补铁储存铁水平难以快速恢复到正常水平，患者HGB恢复正常后极易再次进入IDE甚至IDA状态，少数患者反复发作贫血。静脉铁剂治疗IDA时铁可以越过消化道途径，直接被转运至骨髓合成红细胞或恢复铁储存，可彻底恢复至组织铁充足的状态，再次出现导致铁丢失的因素时，储存铁能够抵御大部分打击，患者不容易直接进入IDA这种危害性更大的疾病状态。静脉铁剂治疗不仅可以改善复发性IDA患者的贫血，还可快速补足患者的铁储备，对于复发性IDA患者似乎是一种更理想的选择。

我们比较复发性IDA患者转换为静脉铁剂治疗和继续口服铁剂治疗的疗效反应。铁剂治疗后1周，静脉铁剂组IDA患者HGB较治疗前基线水平中位增高13（0，23）g/L，口服补铁治疗患者HGB升高迟缓，均发生在治疗1周以后。治疗后4周和8周，静脉铁剂组HGB增长均显著高于口服铁剂治疗组，HGB正常率也显著高于口服铁剂组。治疗后4周血液学反应率在静脉铁剂组即可达到80％，治疗后8周更是接近均能获得疗效反应，显示出良好的疗效。而继续采用传统口服铁剂治疗组，4周血液学反应仅3.3％，虽随着治疗时间延长获得血液学反应患者逐渐增多，但至治疗后8周血液学反应率也仍不足半数。我们的结果表明复发性IDA患者静脉铁剂较口服铁剂治疗有效率更高，且治疗能更快、能获得更好血液学反应，这在需要快速提高HGB的IDA患者，如手术前、妊娠中后期等可能更有优势。

SF是铁状态的重要标志物，虽然SF与巨噬细胞中的组织铁蛋白并不完全相同，但研究表明SF与体内储存铁呈正相关[1]。一篇针对不同国家35个IDA指南的系统性综述认为，单纯性IDA患者治疗后SF应在100～500 µg/L范围内[5]。我们的研究显示，治疗后8周，静脉铁剂治疗组血清SF增长显著高于口服铁剂组，静脉铁剂组一半以上SF>100 µg/L，而口服铁剂组则无一例患者达到SF≥100 µg/L。静脉铁剂治疗不仅可以改善复发性IDA患者的贫血，还可快速补足患者的铁储备。就后者而言，静脉补铁治疗较之口服补铁优势更为明显。

文献报道显示口服铁剂的不良反应可高达30％～70％，而本研究中口服铁剂组不良反应也达到了20％，均为消化道反应，而消化道不良反应是口服铁剂治疗最常见的药物不良反应（ADE），与口服铁剂在肠道内的吸收率较低有关[4],[7]。未被吸收的铁增加肠黏膜中自由基的产生和过氧化，引发肠道炎症，减少肠道的有益共生细菌，增加潜在病原体的丰度，显著降低了患者对治疗的耐受性和依从性[8]。对于病因为消化系统疾病的IDA患者，这些肠道毒性可能进一步加重患者胃肠道疾病[8]。蔗糖铁是全球最常用的静脉铁剂疗法之一，具有良好的疗效和安全性[9]–[11]，其活性成分是氢氧化铁（Ⅲ）-蔗糖复合物，这种结构与生理状态下的SF结构相似，在生理条件下不会释放出铁离子，且吸收率极高，注射后近100％的铁可用于合成红细胞，ADE较少[12]。一项针对超过3 000万剂静脉铁剂的回顾性分析[13]发现，蔗糖铁危机生命ADE（死亡、心脏骤停、昏迷和过敏样反应）的发生率仅为6×10^−7^。一项针对103项铁剂治疗试验的Meta分析[14]发现，静脉注射铁疗法只报道了轻微的输液反应，且与严重ADE或感染的风险增加无关。而本研究中静脉铁剂组仅有2例（3.3％）发生了轻度皮疹，无严重ADE以及输注不良反应，无一例患者因ADE停药，这与许多研究中蔗糖铁的不良反应一致。本研究表明对于复发性IDA患者来说，静脉铁剂的耐受性更好。

复发性IDA严重影响患者生活质量及生存率，但目前对这一群体的研究较少。本研究中发现复发性IDA患者病史大多数较长，中位病史5年，病史长达者可达39年，贫血复发频率高，中位复发次数为2（1，7）次，铁储存量低，SF <15 µg/L即对缺铁具有高度特异性（96％），患者中位铁储存量仅有4.3（1.0，15.7）µg/L[1]，继续选择口服铁剂治疗则意味着更长的口服铁疗程。长期的病史、较高的复发频率和较低的生活质量给复发性IDA患者及家庭带来了沉重的疾病负担，亟需一种更有效的治疗方案降低患者复发率、提高患者生活质量。静脉铁剂能快速提高血红蛋白水平和补足体内铁储备，并进一步减少复发性IDA的发生率，尤其对于无法及时去除病因的患者，可以有效降低IDA复发频率并延长复发时间。充足的铁储备也有助于提高患者的生活质量，减轻疾病负担[3]。静脉铁剂在国内外推荐用于口服铁剂无效或不耐受、需要快速提高血红蛋白水平、口服铁剂不能满足失血损失铁量等情况[2],[12],[15]，对于口服铁剂有效但反复复发的患者来说静脉铁治疗显然也是一种理想的治疗方案。

对于患者和医疗工作者来说，IDA一直没有得到足够的重视。在本研究期间，虽然有大量的患者接受了诊断和治疗，但是能够按照诊疗规范进行血液学和铁代谢随访检查的患者样本数量非常少。我们对复发性IDA进行了静脉铁剂治疗和口服铁剂治疗的回顾性研究，结果证实了静脉铁剂治疗能够更快、更有效地纠正患者的贫血状况，短时间内补充足够储存铁，安全性良好。本研究为非前瞻性、非随机对照研究，缺乏严格的控制条件和随机纳入病例，加之仅依赖单中心数据及样本量较小，这些因素均可能对最终结论产生一定影响。尽管如此，作为初步调查，本研究在指导临床治疗方面仍具有一定参考价值，并为后续研究奠定了基础。
